# Public Health Nurses’ Knowledge and Attitudes Regarding Climate Change

**DOI:** 10.1289/ehp.1104025

**Published:** 2011-11-29

**Authors:** Barbara J. Polivka, Rosemary V. Chaudry, John Mac Crawford

**Affiliations:** Ohio State University, Colleges of Nursing and Public Health, Columbus, Ohio, USA

**Keywords:** climate change, public health nursing, public health services

## Abstract

Background: Climate change affects human health, and health departments are urged to act to reduce the severity of these impacts. Yet little is known about the perspective of public health nurses—the largest component of the public health workforce—regarding their roles in addressing health impacts of climate change.

Objectives: We determined the knowledge and attitudes of public health nurses concerning climate change and the role of public health nursing in divisions of health departments in addressing health-related impacts of climate change. Differences by demographic subgroups were explored.

Methods: An online survey was distributed to nursing directors of U.S. health departments (*n* = 786) with Internet staff directories.

Results: Respondents (*n* = 176) were primarily female, white public health nursing administrators with ≥ 5 years of experience. Approximately equal percentages of respondents self-identified as having moderate, conservative, and liberal political views. Most agreed that the earth has experienced climate change and that climate change is somewhat controllable. Respondents identified an average of 5 of the 12 listed health-related impacts of climate change, but the modal response was zero impact. Public health nursing was perceived as having responsibility to address health-related impacts of climate change but lacking the ability to address these impacts.

Conclusions: Public health nurses view the environment as under threat and see a role for nursing divisions in addressing health effects of climate change. However, they recognize the limited resources and personnel available to devote to this endeavor.

Climate change has been defined as significant and distinct changes in measures of climate that persist for more than a decade [U.S. Environmental Protection Agency (EPA) 2008]. Although climate change can result from natural or man-made factors, the greater contribution in the modern era is from release of greenhouse gases (including carbon) through human activities such as burning fossil fuels; altering land through processes such as deforestation, desertification, and urbanization; and industrial processes (Frumkin et al. 2008; Intergovernmental Panel on Climate Change 2007a, 2007b). Given that the principal contributions to climate change are believed by some to stem from human activities, systematic effective human interventions are needed to arrest and prevent continued climate change. More important, the linkage between climate change and harmful effects for both human health and the environment necessitates responsive action by public health (Sayre et al. 2010).

Climate change affects human health directly and indirectly through such mechanisms as temperature extremes, air pollution, increased allergens, extreme weather events, rising sea levels, and altered patterns of infectious disease vectors [Frumkin et al. 2008; National Association of County and City Health Officials (NACCHO) 2010]. The range of deleterious effects that have resulted and may yet result from unchecked increasing climate change are indicative of the profound threat that confronts our way of life (McFarlane 2010). For example, climate change has the potential to alter normal human development through malnutrition from decreased food supplies and exposure to increased pesticide use and harmful algal blooms (U.S. EPA 2008). Extremes in heat and cold, air pollution, and increased allergens also have heightened health impacts that are particularly threatening to vulnerable populations such as children, older adults, those with preexisting illnesses, and the poor (Frumkin et al. 2008). Others at increased risk of health effects from climate change include those living alone and those living in urban environments already stressed by deteriorating infrastructure, air pollution, and the heat island effect that causes cities to be relatively hotter than nearby rural areas (U.S. EPA 2008). Extreme weather events such as heavy rains, tornados, and droughts; flooding and contamination from sewage and chemicals from sea level rise; and disruptions to the social system such as economic insecurity, homelessness, and conflict affect multiple populations, although their effects also will more profoundly affect those vulnerable groups noted above as well as other socially marginalized groups (Afzal 2007; Frumkin et al. 2008; NACCHO 2010). Like other socioenvironmental factors, such as locating toxic waste sites and neighborhood blight, the health effects of climate change are environmental justice issues that contribute to undue hardship and health disparities among the most vulnerable (U.S. EPA 2009). As safety net providers, public health agencies serve those vulnerable populations that will be most affected by climate change (Bedsworth 2009).

NACCHO urges local health departments to act directly and collaboratively to reduce the severity of health effects from climate change (NACCHO 2010). By definition, public health is embodied in society’s efforts to assure the conditions that support healthy people in healthy communities (Public Health Functions Steering Committee 1994). Public health interventions addressing climate change fall within the basic framework of public health: the 3 core functions (assessment, policy development, and assurance) and the 10 essential services (monitor, diagnose and investigate, inform and educate, mobilize, develop policies and plans, enforce, link, assure, evaluate, and research) (Frumkin et al. 2008). Public health interventions are directed at individuals, families, subpopulations in the community, the community as a whole, and systems within the community. The American Public Health Association (APHA 2011) recommends using a disease prevention model that includes primary (prevent a health event from occurring), secondary (early detection or prompt treatment of a health event), and tertiary (minimize long-term complications or sequelae of a health event) prevention strategies. Primary prevention can include health teaching to increase older adults’ knowledge of the risks of excessive heat. An example of a secondary prevention strategy is immunizing individuals at risk of a waterborne illness that may ensue from contaminated flood waters. Tertiary prevention includes such services as supportive counseling to survivors of a hurricane.

Public health nurses (PHNs), who comprise the largest professional component of the public health workforce, can play an important role in addressing the public health threat and health effects of climate change. Environmental health is an important practice realm for all nurses (Pope et al. 1995) and more specifically for PHNs (American Nurses Association 2007; APHA 2005). As generalist practitioners focusing on the health of populations within the community, PHNs practice with an ecological and holistic perspective that recognizes the environment as a broad determinant of health and well-being (Morris 2010). PHNs are bound by both nursing ethics and public health ethics to address the fundamental causes of disease, prevent adverse health outcomes, and support the implementation of programs and policies in ways that enhance physical and social environments (APHA 2005). PHNs work within their scope of practice to provide such interventions as outreach to inform vulnerable and underserved populations about health issues and services; health teaching of individuals, families, subpopulations, and the community at large; collaboration to address health issues; social marketing to advance knowledge, attitudes, and beliefs about health; advocacy to advance human health and well-being; and policy development and enforcement to effect system change (Keller et al. 2004).

Given the seriousness of the public health threat of climate change and the appropriateness of PHNs’ roles in public health department strategies to address climate change, we sought to determine the knowledge and attitudes of public health nursing administrators (PHNAs) regarding climate change, health effects of climate change, and their public health nursing division’s ability to address climate change. Data reported here are part of a larger study aimed at testing local health department interventions to enhance energy conservation and efficiency in the community to reduce carbon intense living, which contributes to climate change.

## Materials and Methods

This study was approved by the Ohio State University Institutional Review Board with a waiver of informed consent. Data were collected using standard web-based methods (Dillman 2007) from a national survey of PHNAs. The survey frame was PHNAs in all U.S. state and local health departments (*n* = 786) with published Internet staff directories. There are approximately 2,353 U.S. public health departments; however, it is unknown how many have a nursing division. An e-mail invitation was sent to PHNAs in March 2010 explaining the study and providing a link to a secure website (Checkbox Survey; Checkbox Survey Solutions, Inc., Watertown, MA) for completing the anonymous survey. Although the survey was directed at PHNAs, we accepted completed surveys from participants who self-identified as PHNs. The survey took an average of 23 min to complete. Reminder e-mails were sent approximately 1, 2, and 3 weeks after the initial e-mail invitation; 176 surveys were completed (22% response rate). Data were downloaded into SPSS (IBM SPSS Statistics 19; International Business Machines Corporation, Armonk, NY) for analysis.

The web-based survey was created based on existing instruments (Environmental Defense Fund 2008) and on a focus group discussion with PHNAs of Ohio local public health departments (*n* = 6). We report on survey items focusing on climate change knowledge and attitudes, perceptions of health impacts of climate change, and the role of their public health nursing divisions in addressing health impacts of climate change. Eight survey items focused on general knowledge regarding the relationship between humans and environment [see Supplemental Material, Table 1 (http://dx.doi.org/10.1289/ehp.1104025)]. Three items assessed attitudes toward climate change (see Supplemental Material, Table 2). Perceptions of health-related impacts of global climate change were assessed by presenting a list of 12 health issues and asking whether each issue had already increased or would increase within the next 20 years as a result of climate change (yes/no/don’t know). A total health-related impact score was calculated by summing the “yes” responses. Participants also were asked if in the next 20 years, the health-related impacts of climate change would be serious in their jurisdiction, in the United States, and globally (see Supplemental Material, Table 3). The current role and ability of their public health nursing division in addressing health-related impacts of climate change were assessed with four items. A range of –3 (strongly disagree) to 3 (strongly agree) were the response options for the role of the public health nursing division, climate change knowledge, and the locality of health-related impacts. These items were collapsed and recoded as either disagree, neutral, or agree.

**Table 1:** Participant demographics.

Characteristic	*n* (%)
Position	
Public health nursing administrator	114 (79.7)
Public health nurse	29 (20.3)
Sex	
Female	134 (93.7)
Male	9 (6.3)
Race/ethnicity	
White	130 (92.9)
African American	7 (5.0)
Other	3 (2.1)
Hispanic	2 (1.4)
Education	
Associate degree/technical education	25 (17.5)
Baccalaureate degree	62 (43.4)
Masters degree/PhD	56 (39.2)
Age (years)	
29–39	9 (6.4)
40–49	27 (19.3)
50–59	70 (50.0)
60–75	34 (24.3)
Length of time in current position (years)	
< 5	48 (33.8)
5–10	44 (31.0)
> 10	50 (35.2)
Self-reported political views	
Liberal	52 (36.6)
Conservative	46 (32.4)
Moderate	44 (31.0)
Type of health department	
County	97 (69.3)
State	17 (12.1)
District	15 (10.7)
Municipal	11 (7.9)
U.S. region	
West	30 (21.4)
Midwest	54 (38.6)
South	44 (31.4)
Northeast	12 (8.6)

**Table 2:** Perceptions of role of nursing division in addressing health-related impacts of climate change [*n* (%)].

My nursing division:	Agree*a*	Neutral	Disagree*b*
Has a responsibility to address the health-related impacts of climate change		82 (51.3)		38 (23.8)		40 (25.0)
Actions can decrease the health-related impacts of climate change		60 (37.5)		44 (27.5)		56 (35.0)
Has the ability to address the health-related impacts of climate change		35 (21.9)		26 (16.3)		99 (61.9)
Is prepared to address the health-related impacts of climate change		16 (10.1)		23 (14.5)		120 (75.5)
**a**Agree includes strongly agree, somewhat agree, and mildly agree. **b**Disagree includes strongly disagree, somewhat disagree, and mildly disagree.						

Responses were analyzed descriptively. Chi-square analysis and analysis of variance determined differences among these respondent subgroups: position type (PHNA, PHN), educational preparation (associate’s degree, baccalaureate, masters/PhD), age (< 54 years, ≥ 55 years), political perspective (moderate, conservative, liberal), and U.S. region (West, Midwest, South, or Northeast).

## Results

Most respondents were PHNAs, female, and white, had worked in public health for ≥ 5 years, held a baccalaureate degree or higher, and worked in a county public health department (Table 1). The mean ± SD age of respondents was 54 ± 7.8 years (range, 29–75 years). Thirty states and all four regions were represented. Approximately equal percentages self-identified as having moderate, conservative, or liberal political views.

*Relationship between humans and the environment.* Most respondents agreed that humans are severely abusing the environment (75%), plants and animals have as much right as humans to exist (71%), when humans interfere with nature the results are disastrous (69%), the earth has plenty of resources (57%), and the earth is reaching the limit of the number of people it can support (56%) [see Supplemental Material, Table 1 (http://dx.doi.org/10.1289/ehp.1104025)]. More than two-thirds (71%) disagreed with the statements “the balance of nature is strong enough to cope with industrial nations” and “humans will eventually be able to control nature” (68%). Although most (57%) disagreed that “humans have the right to modify the environment to suit their needs,” 35% agreed.

Respondents who self-identified as conservatives were more likely than liberals or moderates to disagree with “we are approaching the limit of the number of people the earth can support” (|^2^ = 9.9, df = 4, *p* = 0.04). Compared with conservatives or moderates, liberals were more likely to disagree with the statements “the earth has plenty of natural resources if we just learn how to develop them” (|^2^ = 13.5, df = 4, *p* = 0.009) and “the balance of nature is strong enough to cope with the impacts of modern industrial nations” (|^2^ = 18.7, df = 4, *p* = 0.001). There were no other significant differences by political perspective, and no significant differences by educational preparation, position type, or region.

*Attitudes toward climate change.* About 46% of respondents indicated that they believed climate change was caused completely by humans, and about 44% responded that climate change was from a balance of human and natural causes[see Supplemental Material, Table 2 (http://dx.doi.org/10.1289/ehp.1104025)]. Respondents who self-identified as conservative were more likely than moderates or liberals to indicate that climate change was a natural phenomenon (|^2^ = 20.5, df = 4, *p* < 0.001). Climate change was considered “bad” by two-thirds and controllable by half of all respondents. Liberal respondents were more likely than conservatives or moderates to consider climate change as bad (|^2^ = 26.9, df = 4, *p* < 0.001) and to regard climate change as controllable (|^2^ = 24.9, df = 4, *p* < 0.001). Respondents with an associate’s degree were more likely to consider climate change as uncontrollable (|^2^ = 12.7, df = 4, *p* = 0.013) compared with those with a baccalaureate or graduate degree. There were no significant differences by position type or region.

**Figure 1:**
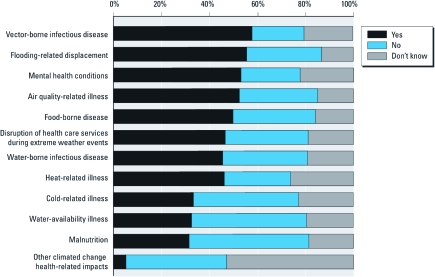
Perceptions of health-related impacts of climate change.

*Health-related impacts of climate change.* More than half of respondents identified vector-borne diseases, flooding-related displacement, mental health conditions, and air-quality–related illnesses as increased because of climate change; however, approximately half did not identify malnutrition and water-availability–related illnesses as affected by climate change (Figure 1). Respondents identified a mean ± SD of 5 ± 3.8 (range, 0–12) health-related impacts of climate change; the modal response was zero impact. PHNAs identified fewer (4.8 ± 3.7) health-related impacts of climate change than did PHNs (6.6 ± 3.9; *F* = 4.7, *p* = 0.03). There were no significant differences by educational preparation, political perspective, or region.

Although most respondents agreed that in the next 20 years the health-related impacts of climate change would be serious in their jurisdiction (65%) [see Supplemental Material, Table 3 (http://dx.doi.org/10.1289/ehp.1104025)], a higher percentage noted that the impacts would be greater in the United States (76%) and globally (81%). Significantly fewer respondents who self-identified as conservative agreed with each statement. There were no other significant differences by subgroups.

*Ability of public health nursing division to address health-related impacts of climate change.* Although approximately half of the respondents agreed that their nursing division has a responsibility to address health impacts of climate change, fewer than 40% felt that their nursing division’s actions could decrease the effects (Table 2). Most respondents noted that their nursing division did not have the ability or were not prepared to address the health-related effects of climate change. Younger respondents were more likely to agree that nursing division actions could decrease health-related impacts of climate change (|^2^ = 7.3, df = 2, *p* = 0.025). There were no other significant differences by subgroups.

## Discussion

This is the first national study assessing PHNs’ and PHNAs’ knowledge and attitudes concerning climate change, perceptions of health-related impacts of climate change, and perceptions of the responsibility and ability of public health nursing to address health-related impacts of climate change. Overall, PHNs and PHNAs view the environment as under threat by human activities. Respondents’ perspectives differed according to political self-identification: Liberals saw more human influence and more negative consequences of climate change, and conservatives saw health impacts of climate change over the next two decades as less serious.

Although most participants recognized the seriousness of health-related impacts of climate change in their own jurisdictions, a greater percentage believed climate change would affect health globally. This finding is comparable to other reports from national surveys in which U.S. respondents were more likely to see climate change as a global rather than a local concern (Akerlof et al. 2010; Leiserowitz 2005). In a national survey of environmental health directors of health departments (*n* = 225), the highest percentage of respondents noted that the impact of climate change would be most serious globally, followed by nationally, and finally locally (Syal et al. 2011). Although the effects of climate change will vary by region and population, the effects will be evident throughout the United States as well as globally (Frumkin et al. 2008). It is crucial that public health professionals as well as the lay public know and plan for the local impacts of climate change.

Knowledge concerning the health-related impacts of climate change by respondents in the present study was minimal, with 19% identifying no impacts and only 4% recognizing all 12 of the listed health issues as potentially affected by climate change. This is consistent with a national public opinion survey by Leiserowitz (2005), in which respondents believed there was no association between climate change and related health effects. Akerlof et al. (2010) reported that 53% of the U.S. public identified increases in epidemics as an outcome of climate change. The impact of climate change on vector-borne diseases was identified in a national survey of environmental health directors (56%) (Syal et al. 2011), local health department directors (80%) (Maibach et al. 2008), respondents in our study (58%), and California local health officers (76%) (Bedsworth 2009). Respondents in the present study and in the study by Maibach et al. (2008) identified disruption of health care services (50%, 53% respectively) and increasing mental health conditions (53%, 41% respectively) as affected by climate change. Air-quality–related illnesses from climate change were noted by almost 80% of California health officers (Bedsworth 2009), by about half of our respondents, and 49% in the findings by Syal et al. (2011). A smaller percentage (44%) of our respondents noted that heat-related illnesses would increase from climate change compared with 73% reported by Maibach et al. (2008). Consistent with our results, the least often identified health-related impacts of climate change by environmental health directors were cold-related illnesses, water-availability illnesses, and malnutrition (Syal et al. 2011), even though these have been recognized in the literature as health effects related to climate change (Costello et al. 2009). Compared with PHNAs, more PHNs in our study identified flood-related displacement, disruption of health care services during extreme weather events, and cold-related illnesses as health-related impacts of climate change. This may relate to the on-site experiences that staff-level PHNs have in providing services to constituents during weather-related events.

Consistent with the findings of Maibach et al. (2008), most respondents to our survey indicated that their public health nursing division did not have the ability or preparedness to address health-related issues due to climate change. Similarly, O’Neill et al. (2010) found that only 37% of local government officials believed excessive heat events were a significant issue for local government. An initial step to engendering PHNs’ sense of professional responsibility regarding the health effects of climate change is through worksite orientation and continuing education for practicing PHNs, as well as content in nursing curricula (Cooney 2011; International Council of Nurses 2008; Kirk 2002).

Although education about the health impacts of climate change is a critical first step, another concern is the role of public health nursing divisions in addressing these health impacts. PHNs need not be experts in climate change to provide targeted interventions such as anticipatory guidance and to participate in public health system adaptations to climate change (Sayre et al. 2010). Guided by the tenets of the Precautionary Principle, the appropriate course for PHNs regarding climate change is to err on the side of caution and support policies and programs that focus on preparedness for climate change through prevention, adaptation, response, and recovery (NACCHO 2010). Leiserowitz (2005) indicated that Americans recognize the reality of climate change but not the urgency of it. PHNAs recognize the urgency of addressing climate change, as reflected by comments of a PHNA participating in our initial focus group: “I do see [climate change] as a problem that needs to be addressed—who knows how long before something big happens.” However, PHNAs in that focus group mentioned that the term “climate change” is controversial. They suggested reframing the discussion to address “sustainability” and linking sustainability to contemporaneous weather and health events to make the issue of climate change acceptable to stakeholders and the public. Others have suggested using language such as “healthy cities programs,” “building resiliency,” or “green cities” (Cooney 2011).

Sheffield and Landrigan (2011) argue that response to climate change should be calibrated to children, a subpopulation shown to be more vulnerable to environmental threats. They note that lowering exposure to particulates and ozone will improve children’s health as well as local community resilience. PHNA focus group participants suggested embedding climate change activities within existing programs such as asthma or obesity prevention. Another subpopulation identified as vulnerable to environmental threats is older adults. PHNs can promote interventions in partnerships with other disciplines and across health sectors for health issues such as heat stroke and respiratory distress due to poor indoor air quality.

There are limitations to this study that must be considered. One limitation is the low response rate (22%). Although this response rate is less than other surveys of PHNAs (52–68%) (Cole et al. 2010; Irwin et al. 2004), those studies were state specific and not national. Our response rate is consistent with the 27% response rate in an online national survey of environmental health directors (Syal et al. 2011). To assess whether response bias was present, demographic information was requested from nonrespondents with the exception of those who specifically requested removal from the sampling frame (*n* = 10). Of the 631 eligible nonrespondents, 103 (16%) provided demographic information. There were no differences between respondents and nonrespondents regarding any demographic characteristics (age, education, race, sex, years worked in public health, political views, type of health department).

Another limitation is the degree of representativeness of the survey frame. Although efforts were made to develop a complete frame of U.S. public health agencies, some health departments were omitted because of inability to locate their online contact information. Finally, although the survey instrument was developed with input from a focus group with PHNAs, was based on another existing tool, and was reviewed by several public health colleagues, there was no formal pilot testing of the instrument.

## Conclusions

It is evident that PHNAs and PHNs see a role for the public health system to address the health effects of climate change despite recognizing the limited resources and personnel available to devote to this endeavor. As state and local governments struggle to balance budgets, present needs take precedence over future liabilities. However, the need to address climate change is here, because the public health threats are real. The question remains: How can scarce resources be allocated for this important public health challenge? Efforts are needed by PHNs to educate, inform, and mobilize the community to develop policies and programs to address the potential consequences of climate change. Future work will build on these findings to explore effective communication strategies regarding these issues.

## Supplemental Material

(152 KB) PDF
