# Supplementary material for: Public Health Nurses’ Knowledge and Attitudes Regarding Climate Change
Source: Environ Health Perspect. 2011 Nov 29;120(3):321–5. doi: 10.1289/ehp.1104025 (PMC3295355; doi:10.1289/ehp.1104025)
Supplement: (152 KB) PDF [file ehp.1104025.s001.pdf]

SUPPLEMENTAL MATERIAL

Public Health Nurses' Knowledge and Attitudes Regarding Climate Change

Barbara J. Polivka
Rosemary V. Chaudry
John Mac Crawford
The Ohio State University
Colleges of Nursing and Public Health
Columbus, Ohio, USA

Table of Content

Supplemental Material, Table 1: Perceptions of the relationships between humans and environment 2

Supplemental Material, Table 2. Attitudes toward climate change 3

Supplemental Material, Table 3. Locality of health-related impacts of climate change 4

Supplemental Material, Table 1: Perceptions of relationships between humans and the environment

	Agree[a]	Neutral	[b]Disagree
Relationship between humans and the environment	N (%)	N (%)	N (%)
Humans are severely abusing the environment.	132 (75.0)	11 (6.3)	33 (18.8)
Plants and animals have as much right as humans to exist.	124 (70.9)	20 (11.4)	31 (17.7)
When humans interfere with nature it often produces disastrous consequences.	121 (69.1)	25 (14.3)	29 (16.6)
The earth has plenty of natural resources if we just learn how to develop them.	99 (57.2)	22 (12.7)	52 (30.1)
We are approaching the limit of the number of people the earth can support.	99 (56.3)	29 (16.5)	48 (27.3)
Humans have the right to modify the natural environment to suit their needs.	61 (34.9)	15 (8.6)	99 (56.6)
Humans will eventually learn enough about how nature works to be able to control it.	33 (18.8)	23 (13.1)	120 (68.2)
The balance of nature is strong enough to cope with the impacts of modern industrial nations.	27 (15.4)	24 (13.7)	124 (70.9)

[a]Agree includes strongly agree, somewhat agree, and mildly agree.

[b]Disagree includes strongly disagree, somewhat disagree, and mildly disagree.

Supplemental Material, Table 2. Attitudes toward climate change

Variable	N (%)
Cause of climate change:	
Human	75 (45.5)
Balance of natural and human	72 (43.6)
Natural	18 (10.9)
Extent climate change is:	
Bad	109 (66.1)
Neutral	46 (27.9)
Good	10 (6.1)
Extent climate change is:	
Controllable	82 (49.7)
Uncontrollable	59 (35.8)
Neither uncontrollable nor controllable	24 (14.5)

Supplemental Material, Table 3. Locality of health-related impacts of climate change

In the next 20 years, the health-related impacts of climate change will be serious in:	Agree[a] N (%)	Neutral N (%)	Disagree[b] N (%)
My jurisdiction	104 (64.6)	30 (18.6)	27 (16.8)
The United States	123 (76.4)	19 (11.8)	19 (11.8)
Around the world	131 (81.4)	13 (8.1)	17 (10.6)

[a]Agree includes strongly agree, somewhat agree, and mildly agree.

[b]Disagree includes strongly disagree, somewhat disagree, and mildly disagree.
